# Sweet Syndrome Associated with Myelodysplastic Syndrome—A Review of a Multidisciplinary Approach

**DOI:** 10.3390/life13030809

**Published:** 2023-03-16

**Authors:** Cătălina Roxana Ferea, Stejara Nicoleta Mihai, Gabriela Balan, Minerva Codruta Badescu, Dana Tutunaru, Alin Laurențiu Tatu

**Affiliations:** 1 Faculty of Medicine, “Carol Davila” University of Medicine and Farmacy, 020021 Bucharest, Romania; 2 Hematology Department, University Emergency Hospital, 050098 Bucharest, Romania; 3 Faculty of Medicine and Pharmacy, ”Dunărea de Jos” University of Galați, 800008 Galați, Romania; 4 Gastroenterology Department, “Sf. Apostol Andrei” County Emergency Clinical Hospital Galați, 800578 Galați, Romania; 5 Department of Internal Medicine, “Grigore T. Popa” University of Medicine and Farmacy, 700115 Iași, Romania; 6 III Internal Medicine Clinic, “Sf. Spiridon” County Emergency Clinical Hospital, 700111 Iași, Romania; 7 Laboratory Department, “Sf. Apostol Andrei” County Emergency Clinical Hospital Galați, 800578 Galați, Romania; 8 Dermatology Department, Clinical Hospital of Infectious Diseases “Sf. Cuvioasa Parascheva” Galați, 800179 Galați, Romania; 9 Multidisciplinary Integrated Center of Dermatological Interface Research MIC DIR, Dunărea de Jos” University, 800008 Galați, Romania

**Keywords:** sweet syndrome, myelodysplastic syndrome, malignancy-associated SS, management, neutrophilic dermatosis

## Abstract

Sweet syndrome (SS) is a rare disease described as a febrile neutrophilic dermatosis with acute onset, the pathogenesis of which has not yet been elucidated. The syndrome is characterized by the sudden onset of erythematous infiltrated papules or plaques located on the upper body and is associated with fever, leukocytosis and neutrophilia. The lesions show a dense dermal infiltration with mature neutrophils. The condition is responsive to systemic steroids. The central nervous system, bones, muscles, eyes, ears, mouth, heart, lung, liver, kidneys, intestines, and spleen may be affected by SS as extracutaneous manifestations. More and more cases have been found to be associated with malignancies, particularly myelodysplastic syndrome, and, less frequently, other hematologic malignancies or solid tumors. Approximately 21% of patients with SS have an associated malignancy and up to 80% of MASS cases are associated with hematological diseases, predominantly myelodysplastic syndrome (MDS) or acute myeloid leukemia (AML). Myelodysplastic syndrome is a clonal disease of the bone marrow characterized by inefficient hematopoiesis, dysplasia of the bone marrow and peripheral cytopenias. Affected patients have a high risk of leukemic transformation. After analyzing later studies and current practical aspects regarding MDS-related SS, we suggest an algorithm for evaluating these patients.

## 1. Introduction

Sweet syndrome was first identified in 1964 by Robert Sweet as a febrile neutrophilic dermatosis with acute onset, with an unclear pathogenesis [1]. He described the cases of eight women with skin eruptions that resembled erythema multiforme, which is associated with leukocytosis, mainly neutrophil polymorphonuclears and fever. The lesions showed dense dermal infiltrations with mature neutrophils and were responsive to systemic corticosteroids. Sweet called the reaction an “acute febrile neutrophilic dermatosis” [2]. Years later, this disease was named Sweet syndrome, and it was established that it can affect the central nervous system, bones, muscles, eyes, ears, mouth, heart, lung, liver, kidneys, intestines, and spleen as extracutaneous manifestations [3].

The syndrome is characterized by neutrophilic leukocytosis associated with fever and erythematous infiltrated papules or plaques located on the upper body: the face, neck and arms [4].

It was initially classified into five groups: classic, parainflammatory, paraneoplastic, associated with pregnancy and secondary to drug administration [1], but it has been categorized by more recent articles into classical SS, malignancy-associated SS (MASS) and drug-induced SS (DI-SS) [5]. Drugs that have been classified as SS inducers are shown in Table 1 [6].

More and more cases have been described to be associated with malignancies, particularly myeloplastic syndrome and, less frequently, other hematologic malignancies or solid tumors [5,7]. Approximately 21% of patients with SS have an underlying malignancy [8]. Some authors consider association with MDS to be more frequent, while others sustain the link with AML, but the majority of articles agree that these two hematological pathologies represent more than 80% of MASS [5]. In 1971, Shapiro et al. described malignancy-associated SS for the first time. MASS can be affiliated as a paraneoplastic manifestation, which can debut before, after or concurrently with the patient’s neoplasm onset. In some cases, skin lesions were demonstrated to be leukemia cutis [9]. When SS is present in MDS, a poor outcome is likely [9]. Other hematological diseases that have been reported to manifest Sweet syndrome are non-Hodgkin lymphoma, chronic lymphocytic leukemia and multiple myeloma [10].

Myelodysplastic syndrome is a clonal disease of the bone marrow characterized by inefficient hematopoiesis, dysplasia of the bone marrow and peripheral cytopenia. Patients have a high risk of leukemic transformation [11].

## 2. Materials and Methods

Using PubMed, we performed a literature search for the term “Sweet syndrome.” As a comprehensive review of SS was published in 2022 by Joshi et al., we selected articles that focus on malignancy-associated Sweet syndrome, with an emphasis on MDS SS. We included articles, case studies and case series from 2011 to the present, but we also included case studies published anterior to 2011 to give proper context. We did not exclude editorials, commentaries, and articles not published in the English language, as some of these give us insight into how consensus was reached for certain subjects. The cases we analyzed are summarized in Table 1. We chose these cases as they represent most common or peculiar presentations for malignancy-associated SS that we identified in the literature. We shall discuss these cases in detail in the “Section 4”.

## 3. Results

### 3.1. Clinical Features

Patients may present with prodromal symptoms such as fever or known infection before skin lesions. These lesions may be followed by fever, arthralgia, generalized malaise or conjunctivitis [12]. Rochet NM et al.’s study reported fever associated with skin lesions in 39% of patients, whereas only some of them had documented fever (38.5 °C). Overall, 27% of patients reported arthralgia that started with the onset of the skin lesions and 27% also reported concurrent fatigue. Only eight study patients had ocular involvement [12].

Additionally, certain autoimmune conditions or dermatological conditions have been observed in these patients, including seronegative rheumatoid arthritis, relapsing polychondritis, pyoderma gangrenosum and Behcet disease [2].

SS is one of the entities within the broader neutrophilic dermatosis’s classification. Neutrophilic dermatoses include SS, neutrophilic eccrine hidradenitis, pyoderma gangrenosum, and Behçet’s disease, among others.

Each disease has some common pathophysiological aspects with an autoinflammatory component (neutrophilic infiltrate), but they are distinguished by particular characteristics such as disease chronicity, type of tissue involved, and clinical presentation. Understanding the pathogenesis is essential for diagnosis and therapy [13].

Variants of the syndrome include the following: the “classic” presentation, which may include symptoms within the upper respiratory tract or gastrointestinal tract, inflammatory bowel disease or pregnancy; the “malignancy-associated” presentation, in which the dermatosis is either the first manifestation of an undiagnosed cancer or a complication in an oncology patient; and the “drug-induced” presentation, when the condition is precipitated by the administration of a dermatosis-associated drug [14].

In addition to the common manifestations, patients may present with pulmonary involvement, a rare complication with only 45 cases reported in the literature and only 1 case published in a dermatologic issue [15].

Sweet syndrome can affect many organs as an extracutaneous manifestation, particularly in patients with a malignancy of an extracutaneous site, which has been reported in approximately 50% of cases [6], or in patients with multiorgan affection [16]. The most common extracutaneous sites are the lungs; symptoms vary from flu-like symptoms in upper respiratory tract infection at disease onset, to acute respiratory distress syndrome. Radiology shows diffuse ground-glass opacities or consolidation and the presence of nodular, reticular or patchy infiltration that might associate effusion [17,18]. Bronchoalveolar lavage examination reveals neutrophilic predominance, and cultures are negative [19]. Sometimes pulmonary involvement is established via transbronchial lung biopsies that reveal interstitial inflammation, neutrophil and occasional lymphocyte, macrophage and eosinophil infiltrates in the alveoli, edema and mild fibrosis [18,20].

In patients with heart involvement, myocardial infiltration with neutrophils has been reported together with aortic stenosis, cardiomegaly, aortitis and coronary occlusive disease [21].

Extracutaneous manifestations involving the central nervous system, locomotor system or internal organs have been reported [13]. Central nervous system involvement, which has been known as neuro-Sweet disease (NSD) since 1999 [22], is rare, described mainly in Asian patients. Encephalitis and meningitis are the most frequent manifestations identified upon neurological examination in NSD. Although NSD is highly responsive to systemic corticosteroids, symptoms frequently relapse. Subsequently, in 2005, a set of diagnostic criteria for neuro-Sweet disease were published: (1) recurrent episodes of encephalitis and meningitis, responsive to systemic corticosteroid therapy; (2) dermatological manifestations consistent with SS; (3) exclusion of cutaneous vasculitis, thrombosis, and uveitis; and (4) HLA-Cw1 or B54 positivity at HLA typing. “Probable” neuro-Sweet disease meets the first three criteria [18,22]. In one case published by Oka S. et al., with the recurrence of NSD, the patient developed SIADH. Both SIADH and NDS improved after second-line treatment, which led to the hypothesis that cytokines such as interleukin IL-6 may play a part in NSD and SIADH [23,24]. Additional neurological symptoms for neuro-Sweet have been reported: aphasia, ataxia, hemisensory loss, focal seizures, hemiparesis or movement disorder [25].

Ocular involvement was also reported in SS in 10–72% of cases [11,18] and usually presents as a mild to moderate conjunctivitis, very responsive to corticoid therapy [21,26,27,28,29].

Malignancy-associated Sweet syndrome may present as a paraneoplastic syndrome in various hematological pathologies, including myelodysplastic syndrome (MDS), chronic lymphocytic leukemia, non-Hodgkin lymphoma, or multiple myeloma [10,18]. The WHO subtypes of MDS associated with SS were MDS with multilineage dysplasia and MDS with excess blasts, type 1 (MDS-EB-1), while from the MDS/MPN category SS, it was reported in association with myelodysplastic/myeloproliferative disease, unclassifiable (MDS/MPN-U). The IPSS risk groups were low and intermediate 1. Transfusion dependency was seen in a small percentages of patients. Progression to “high-risk” MDS also occurred (RAEB 1), but there was no leukemic transformation [2] or a low risk of leukemic transformation.

In both histological variants, Histiocytoid-SS and Neutrophilic-SS associated with MDS, cases were reported of cutaneous lesions that preceded the hematologic diagnosis by approximately 6 months [30]. The mortality rate was higher in Neutrophilic-SS associated with hematological malignancy patients than in Histiocytoid-SS patients, while patients with no complementary hematological malignancy had a better survival rate [31].

Kulasekararaj AG et al. hypothesized that acute non-relapsing SS patients presenting with a single, brief, non-relapsing episode of SS had a trigger prior to the onset of SS (with GCSF being the possible trigger). Chronic relapsing remitting SS was described in patients without a MDS diagnosis at the time of their initial skin eruption. The average time from diagnosis of SS to diagnosis of MDS for these patients was 17 months [2].

It is also important to distinguish between Sweet syndrome and VEXAS syndrome (Vacuoles, E1 enzyme, X-linked, Autoinflammatory, Somatic), an autoinflammatory systemic affliction that tends to develop into MDS, MGUS (monoclonal gammopathy of indetermined significance) or VT (venous thromboembolism) [32]. In a cohort of VEXAS syndrome patients, 88% had skin lesions and 32% turned out to be Sweet syndrome [33].

### 3.2. Diagnostic Criteria

In 1986, Su and Liu published a set of criteria for SS diagnosis [4]. Following the review of data from 38 patients, von den Driesch published a modification of these diagnostic criteria in 1994 (Table 2) [13,18,30], according to which all major and two minor criteria were required for SS to be diagnosed. In 1996, Walker and Cohen proposed separate diagnostic criteria for drug-induced SS [18,34]. As evidenced by the available literature, the diagnosis of SS generally seems to be based on the aforementioned current criteria [13,30]; in some cases, however, the diagnosis is not as straightforward, potentially resulting in delayed diagnosis.

Moreover, published reports show the absence of certain criteria as well as the non-inclusion of some emerging variants, thus highlighting the need for more precise diagnostic criteria [35]. Herein, Nofal A. et al. reviewed the clinical, laboratory, and histopathological aspects of the diagnostic criteria for SS and evaluated their validity in the reported literature and in 40 new patients, subsequently proposing certain modifications of the diagnostic criteria (Table 3) [35]. Additional minor criteria for diagnosis include fever (40–80% of patients), increased erythrocyte sedimentation rate, high values for C-reactive protein, neutrophilic leukocytosis, responsiveness to systemic corticosteroids therapy, and the presence of a chronic inflammatory disorder, such as infection, pregnancy, malignancy, drug exposure, or myeloproliferative disorders [15]. The diagnosis is based on both major criteria and at least two minor criteria [9].

The diagnostic criteria for malignancy-associated SS are identical to those for classic SS, except for the substitution of “an underlying malignancy” instead of “an inflammatory disease, pregnancy, vaccination or infection” in the minor criteria [13].

### 3.3. Morphological Variants

The syndrome is characterized by an abrupt debut with erythematous infiltrated papules or plaques, located predominately on the upper body, face, neck and arms, accompanied by fever and leukocytosis with neutrophilia [4].

One morphologic variant of Sweet syndrome is the neutrophilic dermatosis of the dorsal hands, defined by lesions on the dorsal surface of the hand, but not limited to this area [13,36,37,38]. Another clinical variant of dermatosis, subcutaneous Sweet syndrome, presents as erythematous, tender, subcutaneous and deep dermal nodules [39] that mimic erythema nodosum. The lesions of are mainly identified on the lower limbs [14,40]. Mucous membrane lesions have also been described, which present as oral ulcers and various ocular lesions, such as conjunctivitis [13,41]. Other extracutaneous sites for Sweet syndrome are bones, CNS, intestines, liver, ears, kidneys, lungs, muscles, heart and spleen [6,14,42].

Rochet NM et al. reported in an article that most patients had more than one body area affected by Sweet syndrome, with 86% of patients having lesions on the arms, 56% on the torso, and 55% on the lower extremities. Other areas involved were the head (29%), the neck (25%), and in a small percentage of subjects, the oral mucosa (4%) [12].

The lesions at the time of initial evaluation for this study were erythematous or violaceous plaques (51%), papules (42%), or nodules (32%). Other less common lesions reported were vesicles and pustules [12], and these should be differentiated from drug-induced reactions caused by drugs used for the treatment [43]. The lesions were tender in 43% of patients and the swelling of the affected area was only reported in 16% of cases [12].

Other described lesions were elevated, tender, urticated-like plaques. Some of the lesions had a “nipple-like” raised center. The lesions were scattered on the upper body. Larger, violaceous colored lesions may also be present [2].

Other clinical variants include bullous Sweet syndrome—a rare subtype of SS that can present with blisters, flaccid or tense, on the face, acral surfaces, extremities, and torso. Upon microscopic examination, a dermal–epidermal disjunction can be observed [18,43,44,45,46].

Cellulitis-like SS is a rare SS variant defined by tender, erythematous, edematous lesions, hardly distinguishable from bacterial cellulitis. Differential diagnosis is ensured by negative cultures for pathogenic organisms and a lack of response to antibiotic treatment [18,47,48,49].

Recently, Kroshinsky et al. described necrotizing Sweet syndrome, a variant defined by edematous, erythematous, warm cutaneous lesions with sudden onset, soft-tissue necrosis and deep-tissue neutrophilic infiltration. Infections are absent, the episodes tend to be cyclic and the morbidity risk is high [18,50].

### 3.4. Histopathology

Histopathologic findings include the marked edema of the papillary dermis and dermal inflammatory infiltrate, with a dense, band-like, neutrophilic aspect, with mature neutrophils with leukocytoclasis and the absence of vasculitis [14]. On rare occasions, histopathologic features of leukocytoclastic vasculitis [51] may be present, a secondary vasculitis not specific to SS, but which does not exclude this diagnosis [13,52].

Papillary dermal edema is a common feature in idiopathic or malignancy-associated H-SS, but it is less frequently present in H-SS with MDS than in H-SS associated with other hematological malignancies [31]. The dermal infiltrate was dense or organized in confluent clusters in idiopathic H-SS, while H-SS associated with MDS cases showed a dispersed disposition with histiocytoid cells [31]. A perivascular infiltrate was observed in all idiopathic H-SS cases and H-SS with non-MDS malignancies. Histiocytoid cells are the center element in H-SS, characterized by immunohistochemical staining of CD3, CD68, CD163, MPO, and CD20 [31].

No biopsies of H-SS demonstrated purely subcutaneous infiltrates, although some cases did have an extension of the dermal infiltrate into the subcutis. Similarly, some of the conventional Sweet syndrome cases had at least minor involvement of the subcutis in addition to the dermis, but no cases had purely subcutaneous involvement [53].

Histopathological diagnostic criteria for N-SS involve a diffuse neutrophilic infiltrate of the dermis, edema and presence of neutrophils, as predominant cells in the infiltrate, some with nuclear fragmentation [54,55]. Other cells’ presence has been observed, such as eosinophils, on skin biopsies in classical or drug-induced variants of the syndrome [13] or lymphocytes or histiocytes in rare cases [50,56]. The presence of a perivascular neutrophilic infiltrate may lead to leukocytoclastic vasculitis [15,57], a characteristic aspect of postcapillary venules. This is defined by neutrophils with nuclear dust, extravasation of erythrocytes, necrosis, and granuloma formation, with fibrin deposits in vessel walls [58]. This type of infiltrate is usually limited to the papillary and upper reticular dermis; however, sometimes neutrophils may be identified in the epidermis, with a specific aspect of spongiotic vesicle neutrophils [59] or subcorneal pustules [30]; the neutrophilic infiltrate is not limited to superior layers and can extend into the subcutis or hypodermis, affecting adipocytes and/or septa [60].

In all patients’ biopsies that Raquena L et al. included in their study, there was a neutrophilic inflammatory infiltrate, but the predominance was of mononuclear cells, similar to monocytes or small histiocytes, with circumvolute or kidney-shaped nuclei, acidophilic or vesicular, with evident nucleoli and scant, slightly eosinophilic cytoplasm and showing few isolated neutrophils and nuclear debris [31,50]. Small lymphocytic groups with mature aspects were also described in all biopsy specimens, the majority with a perivascular localization.

Immunohistochemical studies revealed that the majority of the infiltrate cells were positive for CD15, CD43, CD45 (LCA), CD68, MAC-386, HAM56 and lysozyme, which suggests a monocytic–histiocytic lineage. However, unexpectedly, most cells with a histiocytic appearance had intense myeloperoxidase immunoreactivity and CD 66abce. Unfortunately, testing for the presence of CD 163 was not possible at the time. Positivity for CD3 and CD45RO classified most lymphocytes as T cells. Fluorescent in situ hybridization excluded the bcr/abl fusion gene in all studied cases [52]. However, Calvo KR’s work describes histiocytoid cells as immature nonblast myelomonocytic precursors that are MPO-, CD163-, CD33-, CD68- and lysozyme-positive and CD34- and CD117-negative, which differentiates them from cells in leukemia cutis [33].

Thus, it has been established that this unusual histopathologic variant of the syndrome should be addressed. Histiocytoid Sweet syndrome is histopathologically characterized by an inflammatory infiltrate of cells resembling histiocytes or M2 macrophages [61], when in fact immunohistochemical studies proved them to be immature neutrophils [62] or immature nonblast myeloid precursor and myelomonocytic cells [33].

It has been hypothesized that these lesions begin with the release of immature myeloid cells from the bone marrow in early stages, and these cells mature to neutrophils in later stages of the disease [53]. Histopathological differential diagnosis should be established with leukemia cutis [63] and other inflammatory conditions characterized by histiocytes interstitially arranged between collagen bundles of the dermis [52,54,61]. After performing specific MDS-associated fluorescence in situ hybridization studies in cases with known bone marrow cytogenetic alterations, a similarity in the skin infiltrate was found, hinting that the immature myeloid cells in the skin were clonally related to the myeloid malignancy. Vignon-Pennamen MD et al. proposed naming this H-SS variant observed in MDS cases as “myelodysplasia cutis” [61]. The same hypothesis is strongly sustained by Calvo KR’s work [33]. Whether H-SS is more frequently associated with malignancy in comparison with classical SS is still unclear, a result of contradictory data [18,63,64].

In a few rare cases of MDS-associated SS, lymphocytic infiltrates were the first feature observed [9,31]. An initially lymphocytic infiltrate could be attributed to an early timing of the biopsy or the ineffective granulopoiesis in myelodysplastic syndromes. A possible connection between the dysfunction of GM-CSFR and MDS patients with initially lymphocytic SS might be the localization of the GM-CSFR gene on the pseudo-autosomal XY region. This could also explain the fact that all patients with this particular subtype of SS identified so far are male [9,54].

Another unusual case reported was that of a patient with Sweet syndrome characterized by normolipemic xanthoma-infiltrated neutrophilic dermatosis developed with myelodysplastic syndrome (MDS) with single-lineage dysplasia [65].

Histological features in patients with chronic relapsing SS include predominantly neutrophilic infiltrate, and fewer cases showed histiocytic and lymphocytic infiltration as their major histological subtype. The majority showed classical neutrophilic SS. The salient features present in most of the biopsies included mild spongiosis of the epidermis, an intense infiltrate with neutrophils in the reticular dermis, marked leukocytoclasis, the presence of lymphocytes and histiocytes in the infiltrate, and the dilatation of blood vessels with endothelial swelling [2].

### 3.5. Pathogenesis

The initiating factor of SS, with classic SS as the pathomechanism’s prototype, has yet to be established, although the action of certain medications, such as granulocyte colony stimulating factors (G-CSF), all-trans retinoic acid (ATRA) or FMS-like tyrosine kinase-3 (FLT3) inhibitors offer a starting hypothesis in cases of hematologic malignancy [13].

Neutrophils in cellular cultures exposed to serum from SS patients have a decreased apoptosis rate and extended survival, which suggests an increased level of G-CSF and other circulating factors in these patients’ plasma. In solid tumors or hematologic malignancies, clonal cells can produce colony-stimulating factors, which can be an important factor in disease progression. The same phenomenon can be seen in drug-induced SS, from the exogenous use of G-CSF, which further sustains the role of G-CSF in SS [13].

It has also been hypothesized that photosensitivity may play a role in Sweet syndrome. Although the exact pathological mechanism is unknown, the syndrome has been experimentally induced by phototesting [56]. One theory suggests that an isomorphic Koebner reaction is involved; another proposed mechanism associates ultraviolet B radiation with neutrophil activation and epidermal production of tumor necrosis factor-alpha and interleukin 8 [66,67,68,69].

### 3.6. Laboratory

The laboratory findings in SS patients, also included in the diagnostic criteria, are as follows: an elevated erythrocyte sedimentation rate (ESR), positive C-reactive protein and increased number of leukocytes [54]. According to certain studies, in men, the mean hemoglobin level for patients classified as having malignancy-associated Sweet syndrome was 10.5 g/dL versus 11.8 g/dL and 13.0 g/dL in classic Sweet syndrome and drug-induced Sweet syndrome, respectively (*p* = 0.0443). In women, patients with MASS had a mean hemoglobin value of 10.0 g/dL versus 12.8 g/dL and 12.1 g/dL in patients with classic SS and DI-SS, respectively (*p* = 0.0035) [11]. Among the male patients with MASS, 84% had anemia and in women with malignancy-associated Sweet syndrome, 88% had anemia [12].

### 3.7. Cytogenetics and Molecular Assays

FISH assays have proved that neutrophils in SS lesions show the same genetic abnormalities as the underlying clonal population of malignant cells identified in the serum or bone marrow, suggesting the provenience of dysplastic neutrophils in the dermis from the same clone as the myeloblasts [13]. Karyotype abnormalities such as 5/del(5q) are more prevalent among AML patients who develop SS rather than AML patients without SS [18,70].

Japanese patients with neuro-Sweet have been reported to have (HLA)-B54 and Cw1 more frequently at HLA typing, with HLA-B51 negativity related to Bechet’s disease [23].

Two interesting cases were published of two patients with SS and underlying *IDH1*-mutated MDS. Skin biopsies identified *IDH1*-mutant neutrophils, a possible molecular link between the causes of the two syndromes and a definite common target for therapy with IDH inhibitors [63]. FMS-related tyrosine kinase-3 (FLT3) mutations have also been identified in cases with AML associated with SS [18,70] in approximately 39% of patients, and FLT-3 inhibitors are a known SS inducer [13]. The use of a single-nucleotide polymorphism array and next-generation sequencing on these patients revealed FLT-3 gene mutations in bone marrow neoplastic progenitor cells and SS lesional skin biopsies in infiltrating mature neutrophils [13].

There have been reported cases linking SS to Familial Mediterranean fever, an inherited disease in which mutations in the MEFV gene, responsible for the expression of pyrin, are causing the disease [13].

A new clinical trial is underway to determine the genetic architecture of neutrophil-mediated inflammatory skin diseases [71].

### 3.8. Treatment

The pathophysiology of MASS is still not established and there are no guidelines for the treatment of this condition. SS responds to treatment with systemic glucocorticoids and the underlying malignancy benefits from specific treatment [62]. It is important to rule out other conditions such as histoplasmosis and necrotizing skin infections due to the stark differences in treatment—corticosteroids for Sweet syndrome and surgical debridement for an acute necrotizing infection [10].

The management of SS is sometimes reliant on the treatment of an underlying condition, but given the nature of the malignancy, severe symptoms, disease progression and possible non-curable condition, the prompt treatment of skin lesions is essential. First-line treatments for SS include corticosteroids and other agents such as potassium iodide or colchicine. Second-line agents for SS include indomethacin, clofazimine, cyclosporin and dapsone [13,14].

The evolution of cutaneous lesions in cases of H-SS is acute, usually without further relapse or, on rare occasions, chronic with cutaneous relapses [31].

The chronic relapsing remitting SS is resistant to treatment. Most patients must be maintained with a higher dose of prednisolone to avoid recurrency. Response to immunosuppressive therapy in these cases is variable. The treatments associated with complete resolution of the skin eruptions and with no relapses were 5-azacitidine, infliximab and methotrexate, but other agents (dapsone, colchicine, azathioprine, ciclosporin) were mostly disappointing. Glucocorticoids are effective in all patients; however, lower doses inevitable resulted in relapses [2].

Some new approaches to treatment have emerged, such as granulocyte and monocyte adsorption apheresis for patients with recurrent SS, drug-induced SS by Filgrastim or patients with high levels of G-CSF [72], or Adalimumab treatment in patients with recurrent subcutaneous SS [73].

According to Calvo KR’s work, MDS cutis or MDS-associated Histiocytoid SS may respond better to hypomethylating agents than corticosteroids, which is an argument in favor of correctly identifying this SS subtype [33].

## 4. Discussion

Although few cases of neuro-SS have been published in the literature, extracutaneous manifestations of SS and the neurological manifestations as a complication of the disease have been well-established and remain undisputed. Cytopenia associated with MDS may disguise the onset of SS, but the evidence of typical skin lesions should amplify the conduct regarding Sweet syndrome’s diagnosis. Furthermore, as establish by Satoko Oka et al. [23], neurological involvement could follow SS lesions after days, months or years, with patients requiring monthly reassessments.

As described, Sweet syndrome triggered after G-CSF has been well-established. In the case presented by S. Nelis et al. [74] (Table 4), clinical symptoms appeared on the third day of Filgrastim (as part of chemotherapy due to evolution of MDS with excess blast). As expressed by Heath MS et al. [13], there is controversy in classifying this case as drug-induced SS, as the causing drug was administered as treatment for the underlying malignancy, MDS. Moreover, patients that developed MDS-associated SS were proven to have higher plasma levels of G-CSF. In this regard, the debut of SS following Filgrastim treatment is similar to an experimental model recreating the disease. The particularity of S. Nelis et al.’s case can be expressed by ophthalmological involvement with complete remission after one week of treatment, disregarding the progression of MDS. In this regard, we cannot stress enough the importance of periodic reevaluation of patients for extracutaneous manifestation and also to check for disease progression or relapse. It is also advisable to check for associated malignancies in cases where they have not been identified, as the literature reports many cases of SS diagnosed prior to malignancy diagnosis.

Chronology in the diagnostics of SS related to myeloproliferative disorders can be difficult. In case 3 [1], dermatological recurrent episodes showed early leukopenia at first with a shift in complete blood count that occurred after 15 months in association with non-megaloblastic macrocytic anemia and neutropenia.

At admission, patient 4 [15] presented severe anemia and low platelets on CBC, sustaining a very high ESR, all in the context of extensive pulmonary ground glass opacities on CT scan and significant skin lesion development. Interdisciplinary collaboration helped completely evaluate this patient with pulmonary involvement on account of MDS. It is noteworthy that, in this case, the hematological investigations revealed an KMT2A cytogenetic event, traditionally associated with an aggressive clinical evolution and short overall survival. As also pointed out by Astudillo L. et al. [17], pulmonary involvement in SS is the most common extracutaneous manifestation and should, in our opinion, be mandatory when evaluating or reevaluating patients for organ involvement in SS.

Following the intense inflammatory background of SS, severe organic involvement could apply, especially to patients with poor MDS prognosis. The impact of cytogenetic analysis with reference to Sweet syndrome’s evolution is yet to be determined. For the present state of the knowledge, we would like to emphasize the importance of cytogenetic assays in establishing prognosis and risk in MDS, and then establishing necessity and periodicity for reevaluation accordingly.

A new and rare variant of SS, normolipemic xanthomized Sweet syndrome (A. Kamimura et al. [65]), clearly discloses that SS classification is not near to completion, while SS pathogenesis has a few starting theories. This unique case describes a prolonged evolution with adverse outcomes, overlooking the MDS that is affiliated with a good prognosis. As concluded by Joshi et al. [18], SS skin lesions can exert both spatial and temporal heterogeneity. Studies that asses a connection between MDS prognosis and onset of SS could benefit the management of this patients. Additionally, this subtype of SS could be a risk worth evaluating, especially in the Japanese population, as the first case was described in a Japanese patient.

Libby et al. [53] discuss a case of Histiocytoid Sweet syndrome, later diagnosed as MDS-EB1 with IDH-1 mutation. Background MDS was exposed with immunohistochemical stains for IDH-1, with positive markers on lungs and skin specimens. Although a bone marrow biopsy appeared negative, molecular and cytogenetic studies revealed the diagnosis. As molecular assays have reshaped MDS classification and patient management over the years, proving their role, they should also be taken into account in the management of SS patients. Moreover, cytogenetics and molecular assays are essential in MDS evaluation and should also be considered for skin and extracutaneous lesions in patients with malignancy-associated SS. Cytogenetic or molecular abnormalities identified on a skin biopsy in a MDS-related SS patient should be used as a baseline for further monitoring these abnormalities and establishing a prognosis and response to treatment.

Multiple organ failure has various causes. In the case presented (Li et al. [3]), CBC and peripheral blood smear were indicators for MDS diagnosis with 8% circulating blasts. Additionally, histopathology of the skin biopsy raised no issues in relation to SS. Unfortunately, the patient’s status declined quickly, leading to a fatal outcome due to MSOF. The rapid progression of MDS should not be ruled out.

**Figure 1:**
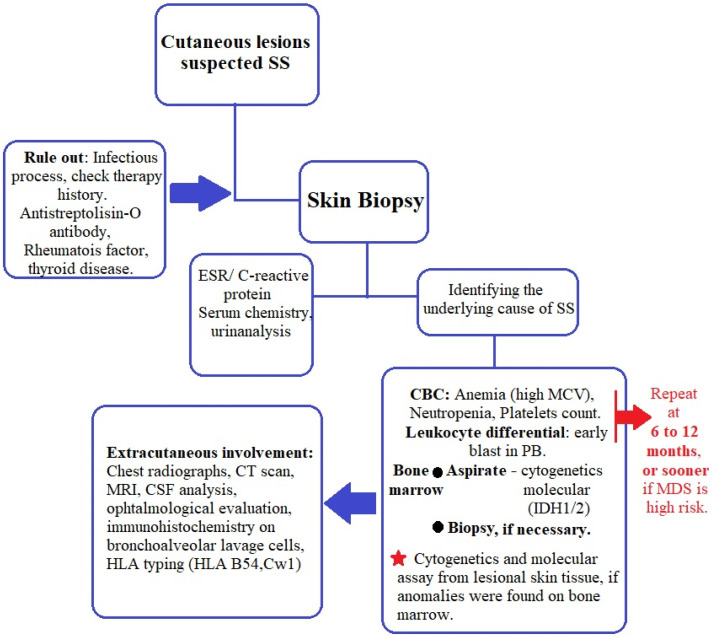
Management of patients with MASS.

The best management plan proposed so far for patients with SS was published by Villarreal-Villarreal et al. [54]. After analyzing later studies and current practical aspects in regard to MDS-related SS, we suggest an algorithm for evaluating these patients (Figure 1). We also encourage visits every 6 to 12 months and CBC and blood smears to be performed, as first proposed by Cohen and Kurzrock [21], with the addition of bone marrow aspirate and bone marrow biopsy when needed to evaluate the MDS status. Cytogenetic and molecular assays should be performed at diagnosis of SS or MDS, whichever is diagnosed first.

Although the etiology of Sweet syndrome is yet to be revealed, it is generally accepted that it has an inflammatory component in all of its variants and presentations. Importantly, this syndrome could be the first manifestation of a malignancy, so each case of SS without an apparent cause should be investigated for one, including hematological evaluation.

## Figures and Tables

**Table 1:** SS inducing drugs.

Antibiotics
Minocycline
Norfloxacin
Quinupristin/dalfopristin
Nitrofurantoin
Ofloxacin
Trimethoprim-sulfamethoxazole
**Antiepileptics**
Diazepam
Carbamazepine
**Antihuman immunodeficiency virus drugs**
Abacavir
Antihypertensives
Hydralazine
**Antineoplastics**
Imatinib mesylate
Bortezomib
Lenalidomide
**Antipsychotics**
Clozapine
**Antithyroid hormone synthesis drugs**
Propylthiouracil
**Colony stimulating factors**
Granulocyte-colony stimulating factor
Pegfilgrastim
Granulocyte-macrophage-colony stimulating factor
**Contraceptives**
Levonorgestrel-releasing intrauterine system
Levonorgestrel/ethinyl estradiol
**Diuretics**
Furosemide
**Nonsteroidal anti-inflammatory agents**
Diclofenac
Celecoxib
**Retinoids**
13-cis-retinoic acid
All-trans retinoic acid

**Table 2:** Diagnostic criteria.

Major Criteria
1. Erythematous plaques or nodules, painful, with sudden debut
2. Neutrophilic infiltrate and absence of vasculitis on histopathological examination
Minor criteria
1. Fever > 38 °C
2. Presence of inflammatory disease, pregnancy, or history of recent upper respiratory infection, gastrointestinal infection, or vaccination
3. Responsiveness to systemic glucocorticoid therapy or potassium iodide
4. Modified laboratory values at presentation (three of the following): a. ESR > 20 mm/h b. Elevated C—reactive protein c. leukocytosis > 8000/µL d. neutrophils > 70%

**Table 3:** Proposed modification to diagnostic criteria.

Constant Features *
1. Clinical: Sudden onset of painful or tender erythematous papules, plaques, or nodules
2. Histopathological: Dense dermal neutrophilic infiltrate
Variable features **
1. Clinical
Fever > 38 °C Atypical skin lesions (including hemorrhagic blisters, pustular lesions, cellulitis-like-lesions)
2. Histopathological
Presence or absence of leukocytoclastic vasculitis Subcutaneous variant Histiocytoid variant Xanthomatoid variant Cryptococcoid variant
3. Laboratory
Elevated ESR Elevated C—reactive protein levels Leukocytosis Neutrophilia Anemia

* Constant clinical and histopathological features must be present to establish a definitive diagnosis. ** Variable features help avoid misdiagnosis of certain cases and may include any new findings yet to emerge.

**Table 4:** Representative cases.

Study	Age	Lesion Type/Location	Clinical Symptoms	CBC	Skin Biopsy with Histopathological Findings	Cytogenetic Analysis	Other Laboratory Findings	MDS Type	MDS Prognostic	∆Time between MDS and SS
Satoko Oka et al. [23]	66	Rash over both legs	fever	WBC-normal; RBC-2.9 mil/mm^3^; Hb 9.2 g/dL, platelets–normal	Neutrophilic infiltration of the dermis and the absence of leukocytoclastic vasculitis, cutaneous vasculitis and thrombosis	46, XX	CRP level: 20.9 mg/dL; HLA-B54 positive/HLA-B51 negative	RA; MDS-SLD	Good	270 days
S. Nelis et al. [74]	46	Conjunctivitis and erythematous nodular rash on lower right eyelid, elbows, forearms and legs	fever	Pancytopenia, Hb 8.1 g/dL; WBC 1550/mm^3^; Platelets 16.000/mm^3^	Dense inflammatory infiltrate, consisting of neutrophils, edema of the papillary dermis and extravasation of red blood cells without sign of vasculitis	N/A	CRP level: 200 mg/L	MDS-EB1/MDS-EB2	N/A	11 days of Chemo/3 days of Filgrastim
F. da Encarnação Roque Diamantino et al. [1]	79	Erythematous, circular plaques, pseudo vesicular, with light pink centers, painful, sized from 0.5 to 3 cm, located on the neck, torso and upper arms	fever	WBC 3.400/mm^3^; non-megaloblastic macrocytic anemia (later state)	Inflammatory infiltrate, dense and with perivascular disposition, consisting mainly of neutrophils, many with leukocytoclasia	N/A	ESR 50 mm/h	MDS-SLD	N/A	0 days
A. Mizes et al. [15]	59	Violaceous papules located on upper and lower limbs	fever	Pancytopenia, Hb 6.0 g/dL, Platelets 30.000/mm^3^	Focal parakeratosis and mild spongiosis overlying a deep dermal, subcutaneous, and peri-eccrine neutrophil rich inflammatory infiltrate	KMT2A—FISH analysis	CRP level: 38.39 mg/L, ESR 140 mm/h	MDS-EB2	Poor	1 day
A. Kamimura et al. [65]	62	Erythema and papules on chest, later on upper extremities	subfever	WBC normal, Hb 8.1 g/dL, Platelets normal	Dense infiltration into the dermis, comprising neutrophils, foamy histiocytes, and leukocytoclastic deposits extending into the mid and deep dermis. Neutrophilic infiltration with leukocytoclasia, numerous xanthomatized cells with large foamy cytoplasm faintly positive for myeloperoxidase (MPO), CD68 (PGM-1), and CD163	N/A	CRP 2.34 mg/dL	MDS-SLD (Mgk)	N/A	60 days
T. J. Libby et al. [53]	50	Erythematous papulonodules on his trunk and extremities	N/A	neutropenia and macrocytic anemia	Lobular and septal panniculitis with mononuclear cells that expressed CD68 and myeloperoxidase, later dermal and subcutaneous HSS	N/A	N/A	MDS with IDH-1 mutation	N/A	60 days
Yun Li et al. [3]	63	Tender erythematous skin lesions on the face, neck, and extremities.	fever	WBC- normal, Hb 5.1 g/dL, Platelets 21.000/mm^3^	Mild subepidermal edema, a diffuse infiltrate of predominantly mature neutrophils, and nuclear dust, no evidence of vasculitis or epidermal involvement	N/A	ESR 80 mm/h	MDS-EB1	N/A	30 days

N/A not available.

## Data Availability

Not applicable.
